# Innovations in animal health: artificial intelligence-enhanced hematocrit analysis for rapid anemia detection in small ruminants

**DOI:** 10.3389/fvets.2024.1493403

**Published:** 2024-11-26

**Authors:** Aftab Siddique, Sudhanshu S. Panda, Sophia Khan, Seymone T. Dargan, Savana Lewis, India Carter, Jan A. Van Wyk, Ajit K. Mahapatra, Eric R. Morgan, Thomas H. Terrill

**Affiliations:** ^1^Department of Agricultural Science, Fort Valley State University, Fort Valley, GA, United States; ^2^Institute for Environmental Spatial Analysis, University of North Georgia, Oakwood, GA, United States; ^3^Department of Veterinary Tropical Diseases, Faculty of Veterinary Science, University of Pretoria, Onderstepoort, South Africa; ^4^Institute for Global Food Security, Queen’s University, University Road, Belfast, United Kingdom

**Keywords:** blood biosensor, support vector machines, hematocrit, image classification, FAMACHA score

## Abstract

Due to their value as a food source, fiber, and other products globally, there has been a growing focus on the wellbeing and health of small ruminants, particularly in relation to anemia induced by blood-feeding gastrointestinal parasites like *Haemonchus contortus*. The objective of this study was to assess the packed cell volume (PCV) levels in blood samples from small ruminants, specifically goats, and create an efficient biosensor for more convenient, yet accurate detection of anemia for on-farm use in agricultural environments for animal production optimization. The study encompassed 75 adult male Spanish goats, which underwent PCV testing to ascertain their PCV ranges and their association with anemic conditions. Using artificial intelligence-powered machine learning algorithms, an advanced, easy-to-use sensor was developed for rapidly alerting farmers as to low red blood cell count of their animals in this way to enable timely medical intervention. The developed sensor utilizes a semi-invasive technique that requires only a small blood sample. More precisely, a volume of 30 μL of blood was placed onto Whatman filter paper No. 1, previously soaked with anhydrous glycerol. The blood dispersion pattern on the glycerol-infused paper was then recorded using a smartphone after 180 s. Subsequently, these images were examined in correlation with established PCV values obtained from conventional PCV analysis. Four separate machine learning models (ML) supported models, namely support vector machine (SVM), K-nearest neighbors (KNN), backpropagation neural network (BPNN), and image classification-based Keras model, were created and assessed using the image dataset. The dataset consisted of 1,054 images that were divided into training, testing, and validation sets in a 70:20:10 ratio. The initial findings indicated a detection accuracy of 76.06% after only 10 epochs for recognizing different levels of PCV in relation to anemia, ranging from healthy to severely anemic. This testing accuracy increased markedly, to 95.8% after 100 epochs and other model parameter optimization. Results for SVM had an overall F1 score of 74–100% in identifying the PCV range for blood pattern images representing healthy to severely anemic animals, and BPNN showed 91–100% accuracy in identifying the PCV range for anemia detection. This work demonstrates that AI-driven biosensors can be used for on-site rapid anemia detection. Optimized machine learning models maximize detection accuracy, proving the sensor’s validity and rapidity in assessing anemia levels. This breakthrough will allow farmers, with rapid results, to increase animal wellbeing and agricultural productivity.

## 1 Introduction

The small ruminant farming industry has seen steady expansion in the past few decades and has become a crucial source of employment and income in family agricultural areas (1). One of the main obstacles limiting the productivity of sheep and goats is infection with gastrointestinal parasites (GIP), resulting in substantial economic losses through diminished weight gain and concomitant impaired production of meat, wool, and milk (2, 3). Furthermore, these parasites constitute significant health hazards, frequently leading to production losses and even mortality of young animals and their mothers during and following parturition through weaning (4).

*Haemonchus contortus*, an ubiquitous blood-feeding GIP of small ruminants worldwide (5, 6), due to its high pathogenicity, including anemia and hypoproteinicity, can significantly decrease the health and production of infected animals (7, 8). Effectively managing this parasite is essential for maintaining the health and economic sustainability of the small ruminant livestock industry (7). Anemia presents substantial health obstacles on a global scale, affecting both human health and the wellbeing and efficiency of large and small ruminants, especially in areas with limited access to veterinary services (9, 10). Furthermore, livestock populations in these underserved areas often experience nutritional inadequacies, and together with a variety of parasitic infections, these additional factors can also have a significant impact on the occurrence of anemia (11). Anemic animals can be saved, requiring interventions such as providing additional nutrients and deworming treatments, but if not treated on time, this condition may lead to death of infected animals. Unfortunately, these interventions are often not available in underserved areas with limited resource farmers.

Considering the above, anemia needs to be addressed in livestock, especially small ruminants, particularly in agricultural areas where these animals are a primary source of nutrition and family income (12). Simple, rapid diagnostic testing for anemia is crucial, as it provides an objective and quantitative measure of health that goes beyond the constraints of subjective symptom evaluation (13).

Two diagnostic tests currently in use are measuring an animal’s packed cell volume (PCV) or using the Faffa Malan Chart (FAMACHA) system of anemia detection (14). The PCV level, which measures the percentage of blood volume occupied by red blood cells (RBCs), is a widely used diagnostic indicator (15), but is not practical for producers, as it requires use of anti-coagulant blood tubes (with blood collected by venipuncture) and specialized laboratory equipment (microhematocrit blood tubes and centrifuge, etc.). Researchers have used this procedure to validate the FAMACHA score chart, which is used to match colors on a laminated card to the color of the lower eyelid conjunctiva of sheep or goats with varying levels of anemia (14, 16). For example, a PCV score above 23% corresponds to FAMACHA scores of 1 or 2 and indicates healthy animals (17). A PCV value between 17 and 23% is considered borderline, corresponding to a FAMACHA score of 3 (treatment may or may not be given based on farmers’ decision), while a PCV value between 12 and 17% is considered as anemic, with a FAMACHA score of 4. A PCV value below 12% indicates severe anemia in small ruminants and corresponds to a FAMACHA score of 5 (16). Both the PCV procedure and the FAMACHA system have been widely adopted worldwide by researchers and farmers, respectively, but both can also be time-consuming and require specialized training for proper use. In addition, matching of color of the eye conjunctiva of sheep and goats can be greatly influenced by environmental conditions (sunny or cloudy days) or person-to-person differences, leading to false negative and false positive diagnoses (16). As an alternative, recent progress has led to the creation of portable, user-friendly instruments that enable swift PCV evaluation, even by lab workers, including students and researchers with limited training, although most of these were developed for point-of-care in human medicine.

There have been significant advancements in optical techniques for measuring PCV in human subjects, particularly using microfluidic settings (18, 19), which allow for direct correlation between the grayscale intensity changes of blood and its PCV values. Nevertheless, these procedures frequently encounter issues due to their susceptibility to changes in ambient lighting, which can lead to distorted outcomes in different operational scenarios. However, recent advancements in the detection of PCV on silicon chips employing impedimetric techniques have demonstrated the potential use of red blood cell suspensions in phosphate-buffered saline instead of whole blood, thereby excluding substantial plasma conductivity influences that could potentially impact precision (20). The complexity of this technology also presents issues for point-of-care (POC) applications (21, 22). Similarly, centrifugal microfluidics have been used to determine PCV levels and complete blood counts on a motorized plastic disk (23), but this approach also has constraints in POC environments due to its reliance on uninterrupted power provision and challenges associated with the disposal of non-biodegradable waste (24, 25).

Functionalized paper strips have emerged as viable platforms for efficient medical diagnostics using blood samples (26, 27), and Berry et al. (28) created a paper microfluidic system that combined vertical and lateral channels to regulate the movement of blood cells. Although this latter procedure is simple, it lacks quantitative precision when compared to traditional laboratory tests and necessitates specific manufacturing processes (28).

In summary, the evaluation of blood hemoglobin levels continues to be a vital method for detecting anemia (29). There are several miniature platforms in support, such as paper-based devices that are currently being developed for this end, such as one from Laha et al. (30), comprising a hemoglobin sensor that is integrated into a smartphone via colorimetric signals detected on a paper strip. While being cost-effective and accurate, this method requires meticulous sample preparation and careful handling of reagents. Additionally, the necessary chemical reagents frequently require refrigeration, which may be limiting, particularly in limited-resource farming areas. Frantz et al. (31) also described a fast and cost-effective method employing a smartphone and a non-reactive lateral flow device to determine PCV levels. Nevertheless, it necessitates the use of high-resolution videography to follow blood flow, which can be expected to be impractical in low-resource environments, but also elsewhere, due to the requirement for specialized equipment and experienced staff.

An assessment of the current POC technologies for PCV determination emphasizes notable difficulties, such as the requirement for intricate strip production, the absence of dependable supply chains for sensitive reagents, and the inadequacy of advanced detection technologies for extreme POC circumstances. In the present investigation, the process, from data collection to analysis of images, which has been modified and compared using four different artificial intelligence-machine learning-based models that were developed by Laha et al. (30), is the use of an inexpensive sensor for determining PCV levels using filter paper strip and smartphone-based image analytics.

This novel method utilizes the basic principles of viscous fingering, in which a less viscous fluid (blood) displaces a more viscous fluid (glycerol), resulting in different interfacial patterns (30). Through the examination of these patterns, the Hausdorff fractal dimensions (also known as fractal dimension analysis) can be calculated to establish a correlation with PCV levels (30). The above technique necessitates only a single image of the blood pattern, obtained using a smartphone camera, greatly streamlining the procedure. This method is suited for rapid, on-site testing by individuals with minimal training since it avoids the requirement for specialist reagents or fabrication stages. It involves use of filter paper soaked in glycerol, an innovation with the capacity to transform the process of identifying anemia in locations with limited access to healthcare, in line with global health goals, by enabling precise and immediate identification of anemia. Point-of-care devices are transforming the management of anemia in humans by allowing timely interventions that can greatly enhance health and output, but there has been very limited work to date on rapid identification of anemia level or development of rapid anemia identification biosensors in domestic or farm animals used for production.

Therefore, the research goal in this investigation was to develop a machine learning model (ML)-supported remote real-time livestock health monitoring system as a tool to assist veterinarians, livestock farmers, and other stakeholders in confirming anemia levels in animals. The specific objectives to achieve this goal were as follows:

- Develop an easy-to-use and efficient image acquisition mechanism to obtain quality data from goats for real-time analyses.
- Compare the various AI–ML-based models to train, test, and validate acquired data for determining animal health status through an optimization approach.
- Develop a real-time remote animal health monitoring system using the best-optimized model.

## 2 Materials and methods

A total of 75 intact male Spanish goats (24 months old; 36–50 kg) were used in this experiment. Although the study involved 75 goats, 1,054 blood pattern images were collected and randomly split into a ratio of 70:20:10, creating training, testing, and validation sets over multiple time points from March 2023 to September 2023, providing a robust dataset for analysis. The validation image dataset was never exposed to any part of the different developed models, either for training or testing. This longitudinal approach allowed us to capture variations over time while adhering to ethical guidelines. All animal use protocols were approved by the Fort Valley State University (FVSU, Fort Valley, GA, USA) Agricultural and Laboratory Animal Care and Use Committee (ALACUC approval number F-T-01-2022). The study was carried out especially to ensure that regulations about animal welfare were followed by reducing any conditions that would cause the experimental goats’ deaths, mainly caused by low PCV values. The goats were allowed to acquire a natural parasitic infection by grazing on grass pasture at the FVSU Agriculture Technology Center farm from March through September 2023.

### 2.1 Sample preparation and packed cell volume (PCV) analysis

Blood samples were drawn weekly from the jugular vein of each goat using sterile needles and collected into K2EDTA-coated vacutainer blood tubes (Avantor, Center, PA) to inhibit coagulation. Samples were transported on ice to the laboratory for PCV analysis. To determine the red blood cell percentage, duplicate micro-PCV tubes were filled with blood from each sample; the tubes were sealed with critoseal clay (Carolina Biological, Burlington, NC), centrifuged at 13,700× *g* for 10 min in a micro-PCV centrifuge (BD Clay Adams Autocrit Ultra 3, BD Diagnostics, Grayson, GA), and the PCV levels measured using a PCV reader.

### 2.2 Development of Whatman 1 filter paper-supported biosensor

**Figure 1:**
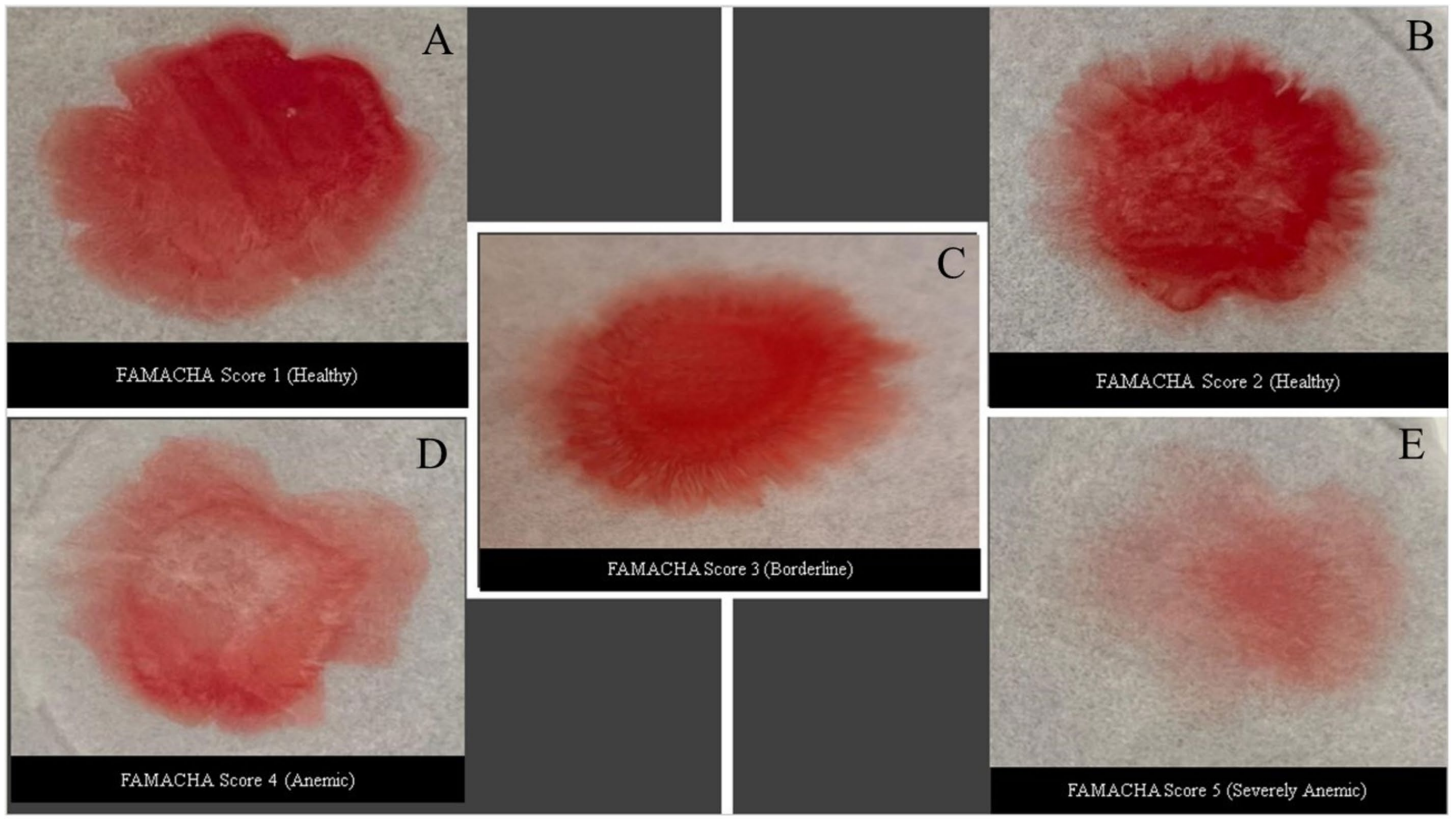
Different blood patterns developed from a drop of blood (30 μL) 180 s after having been placed on glycerol-soaked filter paper after 180 s. Each image was correlated with PCV analysis values and its corresponding FAMACHA score. (A) PCV greater than 28%; (B) PCV range from 22 to 28%; (C) PCV range from 17 to 21%; (D) PCV range from 12 to 17%; and (E) PCV range less than 12%.

The methods described by Laha et al. (30) were utilized to create a rapid, inexpensive, and easy-to-use blood biosensor that can accurately measure PCV levels. However, specific adjustments were made to the experimental procedure to improve its performance. A Whatman 1 filter paper (110 mm in diameter with an average pore size of 11 μm) was used as the detection strip without making any changes to its structure. The filter paper was submerged in anhydrous glycerol (Sigma-Aldrich, St. Louis, MA) for a duration of 5 min and then placed on a firm hollow foundation (open end of a beaker) to function as the test surface. Enough anhydrous glycerol (2 mL) was used to fully saturate the filter paper (30). Anhydrous glycerol was chosen because it has a higher viscosity compared to whole blood, is transparent, biocompatible, and readily available through established supply chains without any specific storage needs. Thirty microliters of blood (same tube of blood used for PCV analysis) were placed onto the glycerol-soaked filter paper from about 1 ~ 2 cm to prevent any splattering. To evaluate the impact of ambient temperature on the sensor’s functionality, experiments were conducted at room temperature (25°C; Figure 1).

### 2.3 Data acquisition and preprocessing

The blood pattern images were taken using a Samsung A54 smart phone with a 50 MP wide-angle primary camera for ensuring high-resolution and quality images for analysis and stored in designated subfolders labeled as “Healthy” goats with PCV values of 22–28% (FAMACHA 2, no treatment needed), “Borderline” (FAMACHA 3; PCV values of 17–21%), “Anemic” (FAMACHA 4; PCV values of 12–17%; requiring anthelmintic treatment), and “Severely Anemic” goats (FAMACHA 5; PCV level of less than 12%; urgently needing treatment). Initially captured in RGB format, these images were first converted to grayscale. This conversion simplified the data by focusing on structural details rather than color variations, which are less relevant for the analysis of blood patterns.

After conversion, the images were subjected to several preprocessing techniques to prepare them for thorough feature extraction and analysis. First, the Otsu Thresholding technique was utilized, which is highly efficient in distinguishing the blood patterns from the background (32, 33). The precise segmentation of the blood patterns enabled a more targeted examination of their essential characteristics. After applying a thresholding technique, the outlines of the blood patterns were detected and accurately delineated with regard to the limits of each pattern (30, 34). Afterwards, the contours were calculated utilizing a minimum bounding rectangle for each pattern, as required to guarantee that each of the subsequent analyses was limited to the specific area of interest (SAI) that contained the blood pattern, hence improving the precision of the feature extraction process to calculate a minimum bounding rectangle for each pattern. This phase is essential, as it guarantees that the subsequent analyses are limited to the SAI that contains the blood pattern, hence improving the precision of the feature extraction process. The data processing and model creation for the study were conducted in Python, employing certain packages to improve performance and reproducibility. Essential libraries comprised ‘numpy (1.21.6)’ for array manipulation, ‘PIL (8.4.0)’ for image processing and preprocessing, and ‘matplotlib (3.5.1)’ and ‘seaborn (0.11.2)’ for data visualization. Deep learning models were constructed utilizing ‘tensorflow (2.11.0)’, and ‘keras’ incorporating an architecture comprised of layers such as ‘Conv2D’, ‘MaxPooling2D’, ‘Flatten’, ‘Dense’, ‘Dropout’, and ‘BatchNormalization’. Data augmentation was executed using ‘ImageDataGenerator’, and the model was refined with the ‘Adam optimizer’, employing ‘ReduceLROnPlateau’ to dynamically modify learning rates. Machine learning models, such as support vector machine (SVM), K-nearest neighbors (KNN), and multi-layer perceptron (MLP), were developed utilizing ‘scikit-learn (1.0.2)’, alongside modules for model assessment (e.g., confusion_matrix, classification_report), data encoding (LabelEncoder), and data partitioning (train_test_split). The incorporation of these libraries and their designated functionality guarantees transparent and reproducible methodologies for image processing, model training, and assessment.

The feature extraction phase utilized two primary methodologies: the Canny algorithm (35) and the Hausdorff Fractal Dimension (HFD) method, sometimes referred to as Fractal Dimension Analysis (FDA) (36). The Canny algorithm functions as an edge detection method, identifying the contours of objects within an image. The process encompasses multiple stages, which include applying Gaussian filtering to smooth the image and diminish noise, calculating intensity gradients, executing non-maximum suppression to eliminate false responses in edge detection, and conducting double thresholding and edge tracking by hysteresis to discern strong and weak edges. In this study, the algorithm was first utilized to identify the borders of blood patterns within cropped areas. This stage is essential for delineating the borders and complexities of blood patterns, facilitating the assessment of more intricate and nuanced aspects that may signify underlying problems (35).

The Canny edge detection algorithm follows a multi-step process to identify edges in an image, and each step involves specific mathematical operations as described below:

Step 1: Gaussian smoothing.

The image used in the study was first smoothed by applying a Gaussian filter to reduce noise. The Gaussian function is given by the following equation below as in Equation 1:


(1) $$G\left(x,y\right)=\frac{-1}{2\pi \sigma^{2}}e^{-\frac{x^{2}-y^{2}}{2\sigma^{2}}}\text{.}$$

where

- *x* and *y* are the coordinates of the image pixel,
- *σ* is the standard deviation of the Gaussian distribution, controlling the amount of smoothing.

Step 2: gradient calculation.

Once the images are smoothed, the algorithm computes the intensity gradients using finite differences to approximate partial derivatives. The gradient in the *x*-direction (Gx) and *y*-direction (Gy) are computed as in Equation 2:


(2) $$G_{x}=\frac{\partial I}{\partial x},G_{y}=\frac{\partial I}{\partial y}$$

where intensity (*I*) is the intensity of the image pixel. The gradient magnitude G and direction theta (*θ*) are calculated as in Equation 3:


(3) $$G=\sqrt{\bar{G_{x^{2}+Gy^{2},}\;}}\theta =\tan\left(\frac{G_{y}}{Gx}\right)$$

Step 3: Non-maximum suppression

In this step, the algorithm suppresses all pixels that are not part of the edges by comparing the gradient magnitude of a pixel to its neighboring pixels along the gradient direction. This results in thin edges.

Step 4: double thresholding and edge tracking

The final step involves applying two thresholds to classify the pixels into strong, weak, and non-edges. Pixels with gradient magnitudes above a high threshold are considered strong edges, while those between the high and low thresholds are weak edges. The weak edges are included in the final edge map if they are connected to strong edges, a process known as edge tracking by hysteresis.

The Hausdorff fractal dimension (HFD) method is a mathematical technique employed to assess the complexity of forms or patterns by the measurement of their fractal dimension. Fractal dimension indicates the variation of detail in a pattern relative to scale, frequently utilized for things exhibiting self-similarity, such as blood patterns. In this particular case for identification of blood pattern, HFD was employed to evaluate the complexity of blood pattern interfaces, offering insights into the extent of fingering or finger-like projections, which denotes the distinctive dispersion of blood patterns. Fingering is a crucial metric for assessing the degree of dispersion, which is closely linked to the severity of anemia. More fingering results in a more distributed blood pattern, which may correspond with more severe episodes of anemia (36). The Hausdorff fractal dimension (HFD), or FDA, is a mathematical tool for analyzing the complexity of a geometrical shape or pattern. It measures how detail in the pattern changes with the scale, making it useful for characterizing self-similar patterns such as blood dispersions.

The Hausdorff dimension DH of a set S is given by the limit as Equation 4:


(4) $$D_{H}=\lim_{e\to 0}\frac{\log N_{\left(\in \right)}}{\log\frac{1}{e}}$$

where

- N(*∈*) (epsilon)(∈) is the number of self-similar structures required to cover the set S at a scale ∈.

In practice, the HFD can be approximated using the box-counting method:

Step 1: grid division.

**Figure 2:**
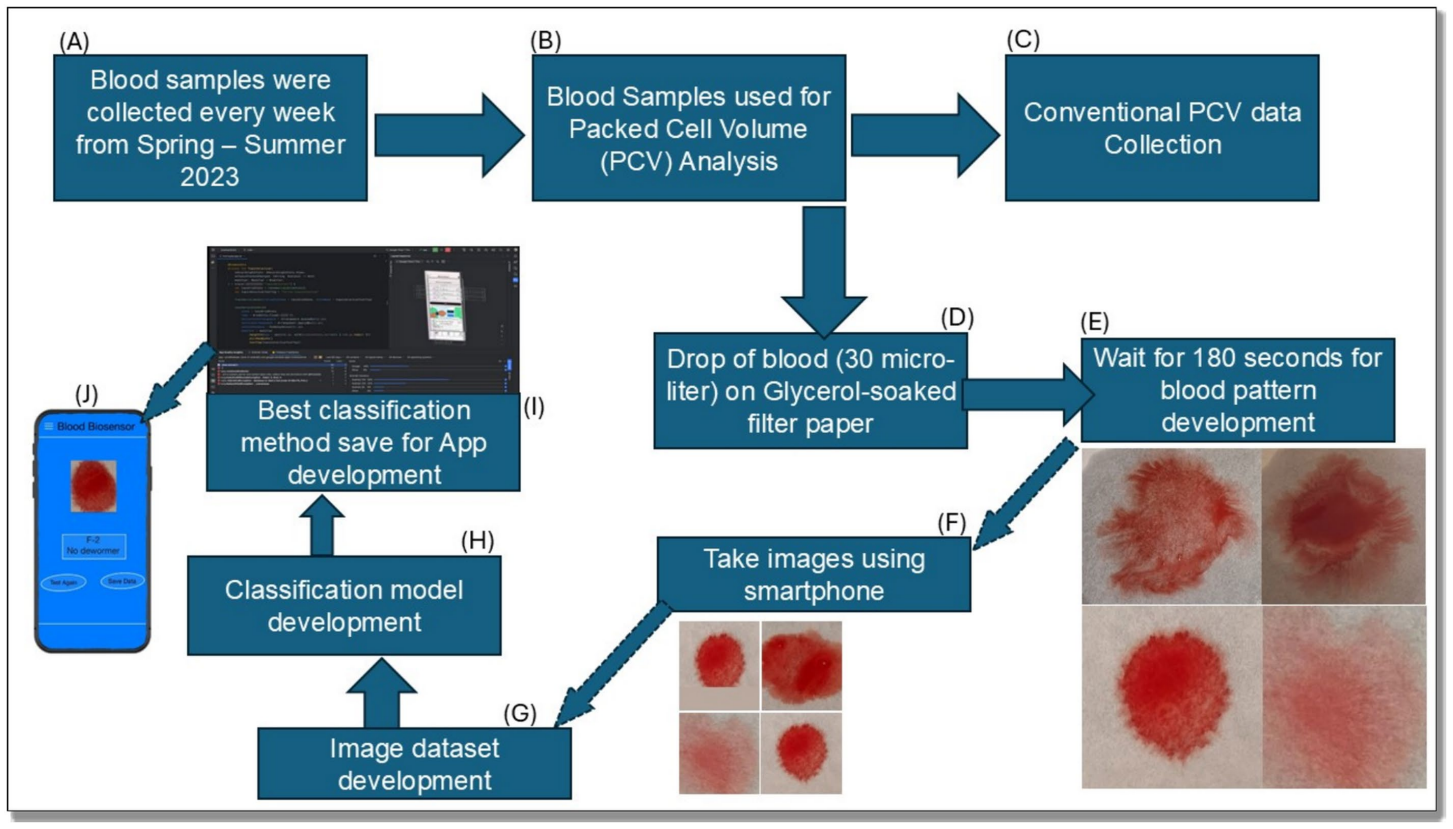
Summary flow diagram for the complete process of development of an anemia detection biosensor and cell phone application. (A) Blood sample collection; (B) PCV analysis; (C) Data collection through conventional process; (D–F) Blood biosensor development step; (G–K) Image dataset development for model and App development.

The image is overlaid with a grid of boxes of size epsilon (Figure 2).

Step 2: counting occupied boxes.

For each box of size ∈, we count how many boxes N(∈) contain part of the pattern.

Step 3: fractal dimension calculation.

The fractal dimension is then estimated by observing how the number of boxes N(∈) changes with different box sizes. The relationship is typically expressed as in Equation 5:


(5) $$N_{\in }\sim \in^{-D_{H}}$$

By plotting logN(∈) against log^−1/∈^, the slope of the resulting line gives an estimate of the Hausdorff fractal dimension DH. A higher value of DH indicates greater complexity or dispersion in the pattern, which is related to the degree of fingering in the blood patterns.

**Figure 3:**
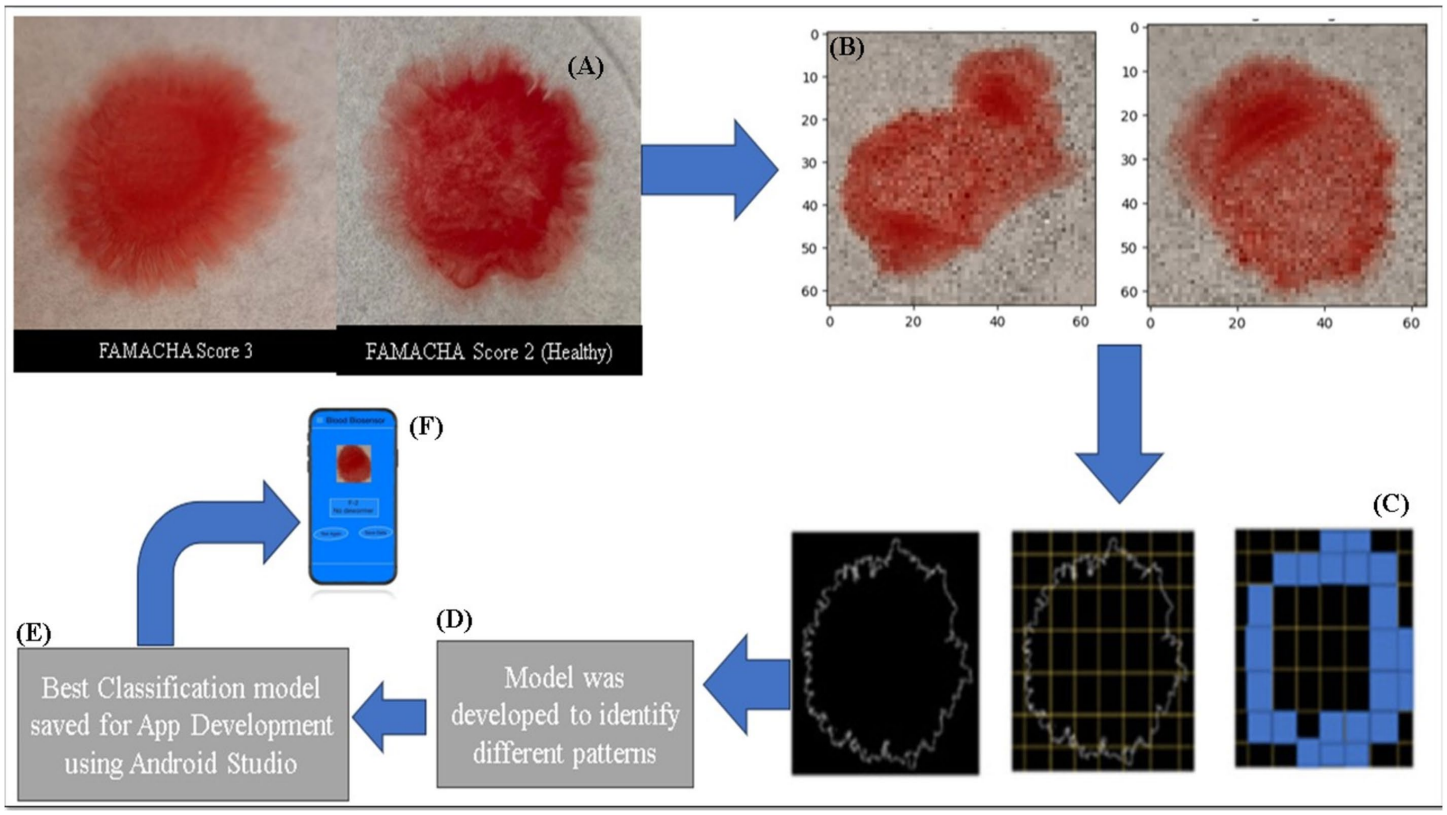
Illustrated images represent the data preprocessing and mobile app development processes, which include normal images, resized images, box counting, classification model development, and smartphone app development (Image modified from (30)). (A) Image Capture: Blood samples were imaged using a smartphone, showing different anemia states (FAMACHA score 3 vs. 2). (B) Image Resizing: Captured images were resized for analysis. (C) Box-Counting: Images were processed using the box-counting method to calculate fractal dimensions, revealing blood pattern complexity. (D) Model Development: Machine learning models classified the anemia severity (healthy, borderline, anemic, severely anemic) based on extracted features. (E) App Integration: The best classification model was integrated into a mobile app for real-time anemia detection.

These mathematical methods are essential in characterizing the complexity of blood patterns, where edge detection identifies the boundaries of the blood and the fractal dimension quantifies the complexity of its dispersion, aiding in the assessment of anemia (Figure 3).

### 2.4 Different supervised machine learning models and training

To examine and categorize the characteristics derived from blood pattern images, a diverse range of machine learning models were utilized, including both supervised machine learning algorithms and a customized deep neural network image classification algorithm designed using Keras.

### 2.5 Support vector machines (SVM)

Support vector machines comprise a type of supervised machine learning technique employed for the purpose of classification, regression, and identification of outliers. The SVM algorithm is widely used in machine learning because of its efficiency in handling both linear and non-linear classification tasks (37). The fundamental concept behind SVM is to identify a hyperplane in a space with N dimensions (where N is the number of features) that effectively separates the data points into various classes (38, 39). Winston (40) uses an analogy of “Fitting the widest possible street” to describe the quadratic optimization problem for hyperplane separations with the algorithm as in Equation 6.


(6) wx+b=0

where *w* is the weight vector, *x* is the input vector, and *b* is the bias term. The hard-margin and soft-margin classification processes determine the maximum distances between different groups to classify the intended pattern or feature from an image (41).

There are numerous hyperplanes that could be selected to distinguish between two classes of data points for binary classification-based problems and more than two classes for multi-classification problems. The goal is to identify a plane that possesses the greatest margin or distance between support vectors, meaning the largest separation between data points belonging to different classes. The SVM algorithm utilizes support vectors (the data points that are closest to the hyperplane). These points are crucial because if they were taken out, the position of the dividing hyperplane would be affected. As a result, SVMs can be regarded as the essential components of the dataset. The strength of SVM lies in its capability to handle non-linear input spaces by employing a kernel trick, which converts the data that are not linearly separable to data that are linearly separable (42).

### 2.6 K-nearest neighbors (KNN)

K-nearest neighbors (KNN) is a straightforward and readily applicable non-parametric, supervised machine learning classifier that uses proximity to make classifications or predictions about the grouping of an individual data point. The KNN algorithm retains a record of all existing cases and categorizes new examples by evaluating their similarity, using measures as distance functions, such as Euclidean, including Euclidean distances, Manhattan, Minkowski, and Hamming distance (43). The objective of the KNN algorithm is to identify the nearest neighbors of a given query point so that a class label can be assigned to that point. The distance functions^1^ enable examining nearby data points by utilizing a majority voting process among these surrounding points. This procedure entails evaluating the neighboring pixels and allocating them to the most suitable category. Each data point is assigned to the class most commonly observed among its K closest neighbors, as determined by the distance function. The selection of K has a direct impact on the precision of the forecasts. While a smaller value of K amplifies the impact of noise on the outcome, a larger one increases the computing complexity. Researchers working with data typically select an odd number when the number of classes is 2. A major limitation of KNN is its substantial decrease in speed in relation to increasing the size of the data being used, as explained in detail by Sidique et al. (44).

### 2.7 Back propagation neural network (BPNN)

Back Propagation Neural Networks comprise a specific kind of ANN that employs the backpropagation algorithm for training. Its primary reputation lies in its efficacy for deep learning models. A BPNN is composed of a minimum of three layers of nodes, i.e., an input layer, hidden layers, and an output layer. Every node, also known as an artificial neuron or perceptron, is linked to weighted connections that are determined through the training process (45). These multilayer perceptrons employ the backpropagation method, which consists of a forward pass where the input data are propagated through the network to produce an output and a backward pass where the error (the discrepancy between predicted and actual output values) is relayed back through the network to adjust the weights. This correction is accomplished using an optimization approach, such as gradient descent. The value of BPNN lies in its ability to learn and represent non-linear and intricate connections. Once trained, it can accurately anticipate outputs when presented with new data, making it very versatile and adaptive in real-world scenarios. Readers can refer to Siddique et al. (42) for more in-depth understanding of the process of BPNN functionality. These models excel at tasks involving pattern identification and were trained using features extracted from the images, such as the computed fractal dimensions (46). The efficacy of these models was evaluated by measuring their accuracy in categorizing the blood patterns associated with various PCV levels.

### 2.8 TensorFlow Keras deep learning model

A customized deep learning model was created utilizing the TensorFlow Keras framework, which included the input layer that is responsible for converting the input image data into a format that can be easily processed by a neural network. The architecture incorporates several layers, with the initial layer consisting of 128 neurons and the subsequent layer consisting of 64 neurons. After each additional layer, there is a further batch normalization layer and a dropout layer. Dropout layers play a critical role in mitigating overfitting, hence improving the model’s capacity to generalize to unfamiliar data (47). The data augmentation procedure was followed as described in Siddique et al. (47). The model concluded with a SoftMax layer that generated a probability distribution for the several categories of PCV levels, enabling accurate categorization. The DL Keras model conducted optimization using the Adam optimizer, with a learning rate of 0.001. The model was trained using the sparse categorical cross-entropy loss function. This approach confirmed that the model not only acquired knowledge efficiently but also delivered dependable estimates across various datasets.

### 2.9 Hyper parameter selection and tuning

In this image classification-based study, we assessed the effectiveness of various machine learning models. The models evaluated included a Keras-based deep learning model, SVM, KNN, and BPNN. We adjusted each model by experimenting with various hyperparameters to find the most effective setup for our blood pattern image dataset.

The dataset consisted of four subdirectories (Healthy, Borderline, Anemic, and Severely Anemic). The images underwent grayscale conversion and were scaled to various dimensions (32, 64, 128, 256, 512 pixels) to evaluate the effect of image size on the performance of the model. The pixel values were standardized, and the fractal dimension was calculated and added as a feature that demonstrated the intricacy of image textures. The class labels were encoded using ‘LabelEncoder’ to transform them into a numerical format that was appropriate for training the model. Principal component analysis was utilized to decrease the dimensionality of the data to 100 principal components, maintaining important variance while decreasing computational complexity.

The Keras model utilized a sequential neural network architecture specifically built for efficient feature extraction and classification. The architecture incorporated a flattening layer to convert the input features into a singular vector, followed by two dense layers with 128 and 64 units, respectively. After each dense layer, batch normalization and dropout layers were added. Batch normalization was used to stabilize and speed up the training process, while dropout was used to reduce overfitting by randomly deactivating a portion of neurons during training. The output layer utilized a softmax activation function to effectively address the multi-class classification task. The model was assembled utilizing the Adam Optimizer, employing sparse categorical cross-entropy loss, and evaluating its performance using accuracy as the metric. The hyperparameters used for tuning were batch sizes of 8, 16, 32, 64, and 128, epochs of 25, 50, 100, 150, and 200, and image sizes of 32, 64, 128, 256, and 512. This resulted in a total of 125 combinations.

The SVM model was examined using various kernel types (linear, radial basis function; RBF) and a range of regularization parameters (C values [0.1, 1, 1.25, 1.5, 2, 2.5, 3, 3.5, 4, 4.5, 5, 10, 20]) to identify the most effective hyperplane that maximized the separation between classes. Therefore, there were 130 possible combinations. The KNN models were optimized by adjusting the number of neighbors, specifically the k values [3, 5, 7, 9, 11, 13, 15]. This resulted in a total of 35 different combinations. The BPNN models were configured using several combinations of hidden layers, namely (128), (128, 64), and (256, 128), along with varied maximum iterations, including 200, 300, 400, 500, and 1,000. This resulted in a total of 75 options.

The performance of each model was assessed using confusion matrices and classification reports, which provided measures such as accuracy, precision, recall, and F1 score. The Keras model employed early halting and learning rate reduction callbacks to mitigate overfitting and improve training efficiency. The outcomes of the hyperparameter tuning process were stored in a CSV (Comma Separated Values) file, and graphical representations were created to compare the performance of different hyperparameters for each model. The most efficient model was stored in TensorFlow Lite format for deployment (48). This strategy guaranteed discovery of the most effective model configurations by systematically adjusting hyperparameters. It utilized both traditional machine learning algorithms and contemporary deep learning approaches to achieve the best possible results for the provided dataset.

### 2.10 Algorithm evaluation matrices

A cost function is a mathematical formula used in machine learning to measure the difference between the predicted output and the actual output (or target). It provides a quantitative representation of how well a model is performing. Lower values of the cost function indicate better performance, as they signify that the model’s predictions are closer to the actual values. Common cost functions include mean squared error (MSE) for regression problems and cross-entropy loss for classification problems (49). For classification tasks, the cost function often penalizes incorrect predictions more heavily, ensuring that the model is adjusted to reduce errors during training (50).

Accuracy is one of the most intuitive performance metrics and is defined as the ratio of the number of correct predictions to the total number of predictions. While accuracy is a useful metric, it may be misleading in cases of class imbalance, where the dataset has a significantly larger number of instances from one class (51). Mathematically, accuracy (%) is expressed as in Equation 7:


(7) $$\mathrm{Accuracy}\;\left(\%\right)=\left(\begin{array}{l} \mathrm{True Positives}+\mathrm{True Negatives}/\\ \mathrm{Total Predictions} \end{array}\right)\times 100$$

Precision can be easily understood as it is the ratio of correctly predicted positive observations to the total predicted positive observations. and it is an important tool to answer the question that: “Of all the instances the model predicted as positive, how many were correct?” Precision is especially useful when the cost of false positives is high. For example, in our image-based veterinary diagnostics, precision is important when false positives (e.g., diagnosing a healthy goat with anemia based on the app output) need to be minimized based on Equation 8 (52).


(8) $$\mathrm{Precision}=\left(\mathrm{True Positives}/\left(\begin{array}{l} \mathrm{True Positives}+\\ \mathrm{False Positives} \end{array}\right)\right)\times 100$$

Recall can also sometimes be understood as sensitivity or true positive rate. It is the ratio of correctly predicted positive observations to all observations in the actual class. It answers the question that: “Of all the actual positive instances, how many did the model correctly identify?” Recall is crucial in situations where minimizing false negatives is critical, such as in disease detection at the locations, where failing to identify a positive case can have serious consequences as in Equation 9 (53).


(9) $$\mathrm{Recall}\;\left(\%\right)=\left(\mathrm{True Positives}/\left(\begin{array}{l} \mathrm{True Positives}+\\ \mathrm{False Negatives} \end{array}\right)\right)\times 100$$

The F1 score is the harmonic mean of precision and recall, providing a balanced measure between the two. It is a better metric than accuracy in cases where class imbalance exists, as it considers both false positives and false negatives. The F1 score is particularly useful in situations where an equal balance between precision and recall is required, such as in classification models where both false positives and false negatives need to be minimized (54).

The formula is as follows as in Equation 10:


(10) $$\begin{array}{l} \mathrm{F1 Score}\;\left(\%\right)=2\times \left(\mathrm{Precision}\times \mathrm{Recall}\right)/\\ \left(\mathrm{Precision}+\mathrm{Recall}\right)\text{.} \end{array}$$

Each model’s effectiveness was assessed using a combination of classification reports and confusion matrices, which highlighted their precision, recall, F1 score, and overall accuracy. The support vector machines (SVM) are exceptionally proficient in high-dimensional spaces and are appropriate for small to medium-sized datasets. Its capacity to manage both linear and non-linear data via kernel functions offers it a flexible option for intricate classification tasks (38, 39).

K-nearest neighbors (KNN) is a straightforward instance-based and efficient method that is simple to implement. The strength of KNN is in its non-parametric characteristics, rendering it advantageous for scenarios where the data distribution remains unidentified (43).

Deep learning models, particularly backpropagation neural networks (BPNN) and Keras-based deep learning models, were selected for their capacity to discern intricate patterns via multiple layers of images, a critical requirement for image classification tasks, including blood pattern analysis (45, 47).

Although convolutional neural networks (CNNs) are efficient for image categorization, they need more extensive, larger datasets for optimal training. Considering that the dataset in this study comprises 1,054 images, simpler ML algorithms such as SVM and KNN may be more suitable for the task. Random Although the random forest models are robust due to the computational complexities or efficiency tradeoffs in real-time scenarios. The best-performing model based on these metrics was selected for deployment and, for practical application, was converted into TensorFlow Lite format, which facilitates its integration into mobile phone-based applications, enabling point-of-care diagnostic capabilities. The complete process for the whole study, including the step-by-step procedures, is illustrated in Figure 2.

## 3 Results and discussion

### 3.1 Box counting method or fractal dimension analysis

**Figure 4:**
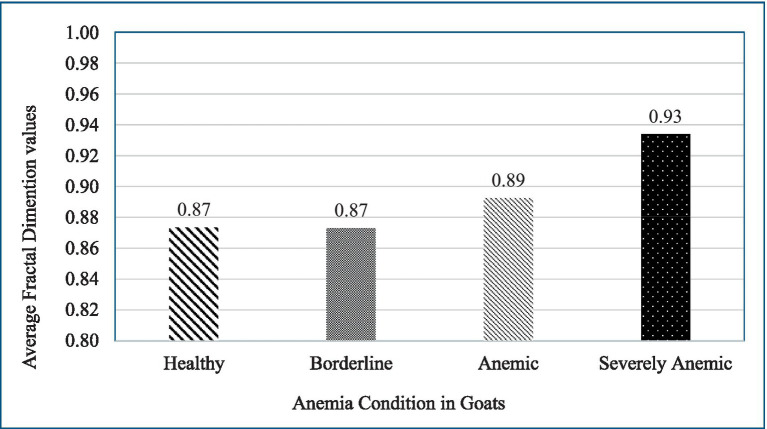
Graph illustrates the changes in the fractal dimension of blood patterns spread on filter paper for goats in varying conditions of health, focusing on anemia. It shows consistent fractal dimensions for healthy and borderline anemic goats (both at 0.87), a slight increase for anemic goats (0.89), and a large increase for severely anemic goats (0.93), highlighting the potential of fractal analysis in diagnosing and assessing the severity of anemia.

Figure 4 represents the average fractal dimension of blood patterns for different anemia conditions, categorized as Healthy, Borderline, Anemic, and Severely Anemic. The average fractal dimension for healthy goats with a PCV value of 22–28% was 0.87, indicating complexity in the blood pattern. This suggests that even in a healthy state, the blood exhibits noticeable textural features, which might be due to its natural viscosity and flow properties.

Individual goats in the borderline category (FAMACHA 3) have a similar fractal dimension of 0.87, i.e., almost identical to that of healthy individuals. This suggests that the early stages of anemia might not significantly alter the textural complexity of the blood patterns, at least not to the extent detectable by this fractal analysis method. There is a slight increase in the fractal dimension to 0.89 for anemic goats. This increase could be indicative of changes in blood composition or behavior as anemia progresses. The blood might begin showing more irregularities or variations in how it spreads on surfaces, reflecting changes in its physical or chemical properties due to reduced hemoglobin levels or other factors.

The fractal dimension for severely anemic goat blood pattern images on average was 0.93. This significant increase suggests a notable change in the textural pattern of the blood, possibly due to more pronounced effects of anemia on blood properties. One possibility is that severely anemic blood might display more pronounced fingering patterns or irregularities, captured as increased complexity or “roughness” by the fractal dimension measurement.

Comparing across the range, there is a clear trend, as the fractal dimension increases with the severity of anemia. This could imply that as anemia worsens, the physical characteristics of blood change in ways that lead to more complex spreading patterns when applied to a substrate like filter paper soaked in glycerol. This increasing trend in fractal dimensions with worsening anemia could result from various physiological changes in the blood, such as alterations in viscosity, the shape of red blood cells, or other factors affecting blood flow dynamics and drying patterns. The graph given above provides a visual representation of how anemia impacts the microscopic appearance of blood, offering a potential tool for easy screening and severity assessment in clinical settings (Figure 4).

### 3.2 Hyper-tuning of different models

The results of the hyperparameter tuning for various machine learning models for image classification tasks are summarized in four tables. Each table highlights the performance of different models with various configurations, providing insights into their accuracy, precision, recall, F1 score, and training/testing time.

The SVM models demonstrated high accuracy with minimal training times (Table 1). For instance, with an image size of 64 × 64 and an RBF kernel with a cost function of 2.5, the model achieved an accuracy of 97.74%, precision of 97.77%, recall of 97.74%, and an F1 score of 97.70%, with a training time of only 0.021 s. This pattern of high accuracy and low training times was consistent across various image sizes, such as 128 × 128, 256 × 256, and 512 × 512, with training times ranging from 0.017 to 0.021 s. The SVM models were efficient in their handling of non-linear relationships through the RBF kernel. The regularization provided by the cost parameter helped in balancing the trade-off between achieving low error on the training data and minimizing the margin, leading to consistently high accuracy across different image sizes. These results highlight the efficiency of SVMs for image classification tasks, making them ideal for quick deployment scenarios for real-time applications and environments with limited computational resources.

**Table 1:** Summary table for the hyper-tuning of parameters for Support Vector Machines (SVM’s).

Model	Image size	Kernel radial basis function	Cost function	Accuracy	Precision	Recall	F1 score	Time elapsed (Seconds)
SVM	32	RBF	4	97.17	97.54	97.17	97.19	0.017
SVM	64	RBF	2.5	97.74	97.77	97.74	97.70	0.021
SVM	128	RBF	2.5	97.74	97.77	97.74	97.70	0.019
SVM	256	RBF	2.5	97.74	97.77	97.74	97.70	0.018
SVM	512	RBF	2.5	97.74	97.77	97.74	97.70	0.019

This table presents the hyperparameter tuning results for the Support Vector Machine (SVM) model with a Radial Basis Function (RBF) kernel for classifying blood patterns in small ruminants to detect anemia levels. “Image size” refers to the pixel dimensions of the images used for classification. “Cost function” represents the penalty parameter that controls the margin trade-off, with higher values corresponding to stricter boundaries between classifications. Accuracy, precision, recall, and F1 score are performance metrics represented in percentage that assess the model’s classification efficiency, while “time elapsed” indicates the total time taken to train the model in seconds. The F1 score is the harmonic mean of precision and recall, providing a balanced measure of the model’s ability to classify data correctly.

The KNN models showed slightly lower accuracy, as shown in Table 2. For example, with an image size of 32 and 3 neighbors, the model achieved an accuracy of 94.91%, precision of 95.41%, recall of 94.91%, and an F1 score of 95.08%. This accuracy remained consistent across different image sizes (64, 128, 256, and 512) with slight variations in precision and recall, indicating that the number of neighbors is a critical factor for KNN’s performance. While KNN is straightforward to implement, its performance is less robust for high-dimensional data compared to other models, which limits its effectiveness for more complex image classification tasks. The KNN models’ slightly lower accuracy is due to their reliance on distance metrics, which can be less effective in high-dimensional spaces. The consistent accuracy across different image sizes indicates that KNN’s simplicity comes at the cost of reduced performance in more complex scenarios.

**Table 2:** Summary table for the hyper-tuning of parameters for K-Nearest Neighbor (KNN) algorithm.

Model	Image size	Number of neighbors	Accuracy	Precision	Recall	F1 score
KNN	32	3	94.91	95.41	94.91	95.08
KNN	64	3	94.35	94.88	94.35	94.05
KNN	128	3	94.35	94.88	94.35	94.05
KNN	256	3	94.35	94.88	94.35	94.05
KNN	512	3	94.35	94.88	94.35	94.05

Table 2 summarizes the performance of the K-nearest neighbors (KNN) model using different image sizes, with the number of neighbors fixed at 3. Accuracy, precision, recall, and F1 score are performance metrics represented in percentage that assess the model’s classification efficiency, while “time elapsed” indicates the total time taken to train the model in seconds. The F1 score is the harmonic mean of precision and recall, providing a balanced measure of the model’s ability to classify data correctly.

The BPNN models exhibited high accuracy and efficient training times (Table 3). For instance, with an image size of 512, neuron configurations of 256 and 128, and 300 iterations, the model achieved an accuracy of 98.30%, precision of 98.31%, recall of 98.30%, and an F1 score of 98.30%, with a training time of 1.53 s. Other configurations, such as an image size of 256 with neurons of 128 × 64 × 200 iterations, achieved an accuracy of 97.74% and a training time of 2.14 s. These results highlight the flexibility of BPNNs in achieving high accuracy with efficient training times through appropriate neuron configurations and iterations. The BPNN models achieve high accuracy with efficient training times by leveraging flexible neuron configurations and iterations. The use of backpropagation and iterative optimization techniques ensures the model converges to a good solution, capturing complex patterns in the data effectively. This balance between complexity and efficiency makes BPNNs a strong contender for image classification tasks, particularly when training time and computational resources are moderate.

**Table 3:** Summary table for the hyper-tuning of back propagation neural network (BPNN) parameters.

Model	Image size	Number of neurons	Iterations	Accuracy	Precision	Recall	F1 score	Time elapsed (seconds)
BPNN	32	(256, 128)	1,000	97.17	97.22	97.17	97.16	3.60
BPNN	64	(128,)	400	96.61	96.67	96.61	96.59	3.00
BPNN	128	(128, 64)	200	97.17	97.27	97.17	97.20	2.60
BPNN	256	(128, 64)	200	97.74	97.82	97.74	97.72	2.14
BPNN	512	(256, 128)	300	98.30	98.31	98.30	98.30	1.53

Table 3 presents the results of back propagation neural networks (BPNN) across various configurations based on the hyperparameter configurations for image classification tasks. “Image size” refers to the dimensions of the blood pattern images in pixels, “number of neurons” indicates the number of units in the hidden layers of the neural network, “iterations” specifies the number of training cycles, Accuracy, precision, recall, and F1 score are performance metrics represented in percentage that assess the model’s classification efficiency, while “time elapsed” indicates the total time taken to train the model in seconds. The F1 score is the harmonic mean of precision and recall, providing a balanced measure of the model’s ability to classify data correctly.

The Keras deep learning model displayed exceptional performance across various configurations (Table 4). For example, using an image size of 32, batch size of 8, and 150 epochs, the model achieved accuracy, precision, recall, and F1 scores all around 98.30% and a training time of 28.85 s. Similar high performance was observed with an image size of 128, batch size of 8, and 100 or 150 epochs, achieving the same high accuracy and slightly different training times (27.51 s). These results illustrate the model’s robustness and ability to maintain high accuracy across different configurations. Additionally, an image size of 256 with a batch size of 8 and 50 epochs maintained a high accuracy of 98.30%, while reducing the training time to 20.87 s. This demonstrates the model’s efficiency in achieving optimal performance through appropriate parameter tuning. The Keras deep learning model’s high performance, with accuracies reaching up to 98.30%, can be attributed to its ability to learn complex patterns through multiple layers of abstraction. Techniques such as dropout and batch normalization help in regularization and stabilization, preventing overfitting and ensuring the model generalizes well to new data. The adaptive learning rates further enhance the model’s ability to converge efficiently.

**Table 4:** Summary table for the hyper-tuning of parameters for simplified Keras deep learning image classification model.

Model	Image size	Batch size	Epoch	Accuracy	Precision	Recall	F1 score	Time elapsed (seconds)
Keras	32	8	150	98.30	98.35	98.30	98.30	28.85
Keras	64	8	150	97.17	97.19	97.17	97.15	29.17
Keras	128	8	100	98.30	98.35	98.30	98.29	27.51
Keras	128	8	150	98.30	98.35	98.30	98.29	27.51
Keras	256	8	50	98.30	98.35	98.30	98.29	20.87

Table 4 presents the results of various Keras model configurations used for image classification in the study. “Image size” refers to the dimensions of the blood pattern images in pixels, “number of neurons” indicates the number of units in the hidden layers of the neural network, “iterations” specifies the number of training cycles, Accuracy, precision, recall, and F1 score are performance metrics represented in percentage that assess the model’s classification efficiency, while “time elapsed” indicates the total time taken to train the model in seconds. The F1 score is the harmonic mean of precision and recall, providing a balanced measure of the model’s ability to classify data correctly.

Comparing these selected models revealed that Keras and BPNN models achieved the highest accuracy, with Keras being slightly more consistent across different configurations. For instance, Keras models consistently achieved accuracy above 98% across different image sizes and batch sizes, while BPNNs achieved similar high accuracy with larger image sizes and specific neuron configurations. The SVM models offered a great balance between accuracy (around 97.74%) and extremely low training times (below 0.021 s), making them suitable for scenarios where quick model deployment is needed. The KNN models, while easy to understand and implement, fell behind slightly in accuracy (around 94.91%) compared to the other models. Each model’s performance was influenced by its inherent strengths and how well it leveraged the characteristics of the image data, guiding the choice of model based on specific needs for accuracy, computational efficiency, and interpretability in different practical applications.

### 3.3 Comparison of different models

**Figure 5:**
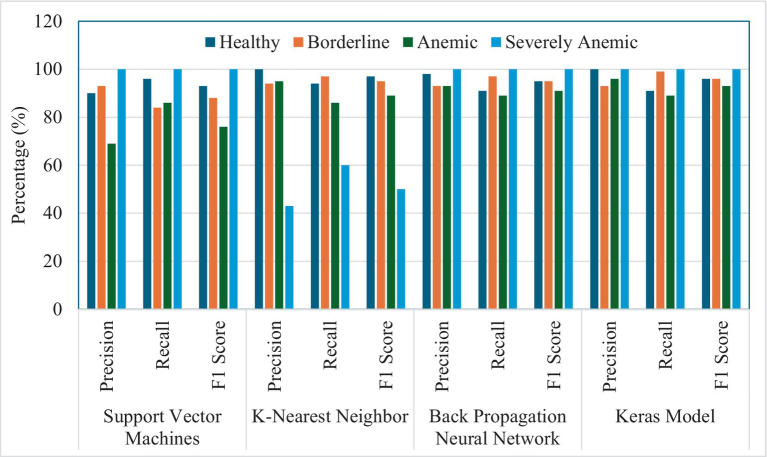
Summary graph representing percentage precision, recall, and F1 score for the ability of four models (SVM, KNN, BPNN, and Keras model) to detect different levels of anemia in goats.

Figure 5 presents a comprehensive performance assessment of four different machine learning models: SVM, KNN, BPNN, and a Keras-based deep learning model. The evaluation was conducted across four diagnostic categories applicable to goats: Healthy, Borderline, Anemic, and Severely Anemic. These metrics are essential for evaluating the suitability and effectiveness of each model in clinical diagnostics, with particular emphasis on the accuracy, recall, and F1 score for each condition. The developed SVM model performed well, achieving a precision of 90% and a recall of 96%, leading to an F1 score of 93%, demonstrating the robustness of SVM model in accurately classifying samples as Healthy. For the borderline condition, SVM demonstrated a precision rate of 93% and a recall rate of 84%, resulting in an F1 score of 88%. This indicates a commendable but somewhat less dependable performance in borderline cases as compared to healthy goats. In the identification of anemic condition or FAMACHA 4 class (Anemic), SVM exhibited a precision rate of 69%, which is relatively lower but compensates with a higher recall rate of 86%, leading to an F1 score of 76%. This indicates a potential confusion with other categories, likely caused by similar symptoms in the feature space. In extremely Anemic goats (FAMACHA 5), SVM demonstrates a perfect performance in all measures (100% precision, recall, and F1 score), demonstrating exceptional model accuracy in identifying evident instances of severe anemia. In a study conducted by Rezatofighi and Soltanian-Zadeh (55), the SVM model successfully classified 400 blood smears with a recognition rate of 90.0%. On the other hand, in our study, SVM was able to identify different anemic conditions with a varying accuracy ranging from 74 to 100%, with an average F1 score of 81–100%. For the validation of the developed models, we have used an unknown validation dataset that was never the part of images used in the training or testing process. Sensitivity, also known as recall of the model, measures the proportion of true positives that were correctly identified by the model. This metric is particularly important in scenarios where minimizing false negatives is crucial, such as medical diagnoses or detection, where failing to identify a condition could lead to severe consequences. The validation image dataset showed that SVM performed moderately well in terms of sensitivity, but its sensitivity for anemic animals (0.88) (Table 5) lagged as compared to BPNN and KNN. This could indicate that the SVM’s linear decision boundaries may not be flexible enough to capture all the variations in the feature space for this class.

**Table 5:** Comparison of different models on validation dataset for different performance metrics.

Metrics	Condition	SVM	KNN	BPNN	Keras
Sensitivity (recall)	Healthy	85	95	95	77
	Borderline	94	94	96	83
	Anemic	88	92	93	92
	Severely Anemic	100	100	100	33
Specificity	Healthy	97	97	99	97
	Borderline	91	97	98	97
	Anemic	99	99	99	84
	Severely Anemic	98	98	98	92
Negative predictive value	Healthy	97	99	99	100
	Borderline	93	93	96	83
	Anemic	96	97	97	97
	Severely Anemic	100	100	100	99
ROC-AUC	Healthy	99	98	99	97
	Borderline	98	99	99	92
	Anemic	99	99	95	97
	Severely Anemic	99	99	99	98

Specificity measures the proportion of true negatives that were correctly identified. High specificity is crucial in scenarios where false positives are undesirable and could lead to misdiagnosis. The SVM model results for the validation dataset showed a strong specificity, with scores ranging from 0.91 to 0.99 across all tested classes. This indicates the model was effective at identifying negative instances and minimizing false positives. Negative precision values (NPV) ranging from 0.93 to 1.00 for the SVM model demonstrated a strong performance in correctly predicting negative instances. Area under the receiver operating characteristic curve (ROC-AUC) measures the area under the ROC curve, which plots the true positive rate (sensitivity) against the false positive rate (1 – specificity) at different threshold settings. It provides an overall measure of the model’s ability to distinguish between positive and negative classes. Based on the ROC-AUC analysis, the SVM model demonstrated excellent ability to distinguish between classes, with values above 0.98.

Although the KNN model demonstrated excellent effectiveness in most settings, achieving precision and recall rates above 90% for the Healthy, Borderline, and Anemic animals, it performed noticeably less functionally in identifying animals in the ‘Severely Anemic’ category. The precision and recall rates were 43 and 60%, respectively, thus resulting in an F1 score of 50%. This decrease in performance may be attributed to KNN’s susceptibility to the selection of features and distance measures in datasets with severe skewness. On the other hand, KNN models showed a precision accuracy of 100% for identifying Healthy animals, Borderline animals at 94%, and Anemic group animals at 92%, as compared to the study conducted by Young (56), using clustering and distance measuring approach, which showed an accuracy of identifying blood cell images with an accuracy of 92.46%. The KNN model performed well in terms of sensitivity, achieving high recall values across all classes (Table 5). Particularly for severely anemic animals (FAMACHA score 5), KNN achieved perfect recall (1.0), which indicates its effectiveness in identifying all instances of this class. The KNN model also showed strong specificity, with values between 0.97 and 0.99, suggesting it was able to correctly identify negative cases and minimize false positives. For the validation dataset, the KNN model was highly reliable at predicting negative instances, with a NPV value of 1.0, ensuring that most of its negative predictions were accurate. The ROC-AUC values for analysis of the validation set using the KNN model were high across all classes, as similar to SVM, indicating a strong ability to distinguish between classes. In another study conducted by Bikhet et al. (57), the KNN model was used with an entropy-based repeated threshold method to divide white blood cell type and classified the images with an accuracy of 90.14%.

The BPNN model in our study exhibited a strong and reliable performance, especially in the ‘Severely Anemic’ animal category, by achieving perfect scores comparable to SVM, as evidenced by its ability to recognize positive samples, namely its consistently high recall rates, which consistently exceeded 89% across all situations.

The BPNN model outperformed all the other models in terms of sensitivity for the validation image dataset, achieving nearly perfect recall for all classes (Table 5). Its ability to correctly identify all positive instances for severely anemic animals (1.0) and other classes demonstrates the model’s effectiveness in minimizing false negatives, along with the high specificity values ranging from 0.98 to 0.99. The BPNN model used for the validation dataset showed NPV values closer to 1.0, suggesting correct prediction of negative instances. With the highest AUC scores across all classes, the BPNN model showed exceptional ability to distinguish between different classes.

**Figure 6:**
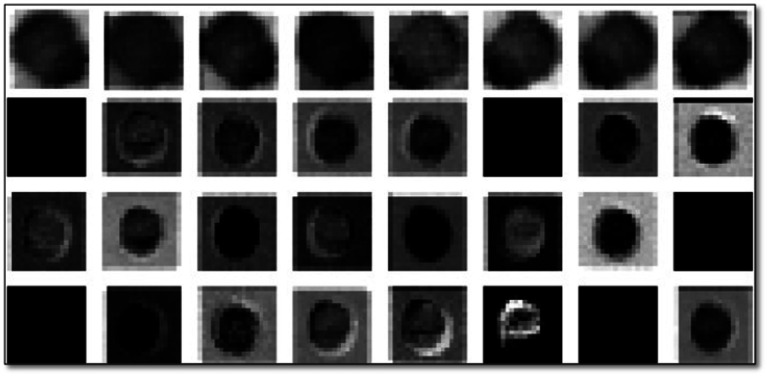
Illustrative examples of trained images from max pooled layer for different blood patterns from goats varying in level of anemia.

In the case of the Keras model developed the deep learning image classification model with batch normalization and adjusted dropout method in conjunction with adjusted learning rate, a perfect precision of 100% and recall rates of 91% were demonstrated for the Healthy and the Severely Anemic categories, having both precision and recall rates of 100%. It was further supported by its ability to efficiently comprehend intricate patterns through the utilization of advanced deep learning techniques (Figure 6). The Keras model showed poor sensitivity, particularly for the class of severely anemic goats, where it achieved a recall of only 33% due to the limited number of validation dataset images. This indicates that the model missed many true positives, especially in underrepresented or more difficult to classify classes. While the model achieved perfect specificity for severely anemic animals, it showed lower specificity for borderline animals (0.84) and anemic animals (0.92), indicating that the model needs a larger image dataset for validation to correctly classify negative samples for these classes. The Keras model achieved lower NPV values of 0.83 for borderline animals. The AUC values for the Keras model were lower than those of the other models, particularly for borderline animals (0.92), indicating that it struggled more to distinguish between this class and others. Liang et al. (58) used a CNN-RNN (convolutional neural network–recurrent neural network) framework to classify blood cell image types and found that their proposed model and compared models were able to achieve a classification accuracy ranging from 84.08 to 90.79%, while our proposed Keras model achieved a precision accuracy for different anemic groups ranging from 90 to 100% based on the extracted image features.

A comparison of different models highlights specific advantages and disadvantages of relevance to different clinical situations in animals. The fact that SVM and BPNN models exhibit exceptional accuracy in identifying cases of severe anemia makes them very dependable for situations where precise diagnosis is crucial to prevent false negatives as regards anemia, which can have serious repercussions for both sheep and goats. The heterogeneity of KNN underscores the potential difficulties in fine-tuning parameters and scaling features, both of which are crucial for enhancing its performance in imbalanced classes. On the other hand, the Keras deep learning model demonstrates the benefits of deep neural networks in processing high-dimensional data and learning non-linear connections, and it is well suited for complex diagnostic tasks that require differentiation between multiple conditions.

Taking into consideration that the Keras model regularly demonstrated a reliable performance across all measures, it is suitable for practical deployment in situations where accuracy and dependability are of utmost importance. Moreover, the utilization of advanced optimization and regularization approaches may enhance the ability of this model to generalize effectively to unfamiliar data, while simultaneously mitigating the risk of overfitting.

The present research examined the merits and drawbacks of various machine learning models, offering a fundamental comprehension essential for choosing the most suitable one according to unique classification requirements. After the training, testing, and validation of the best-performing model, based on performance matrixes, robustness, and observed results, the Keras model developed for the identification and classification of different levels of anemia in goats was used to develop a smart phone application for field trials. The mobile application, *AniHealth*, was developed using Android Studio software (Version: 2022.2.1, Flamingo), which constitutes a Java platform (47).

By deploying the model directly on mobile devices, farmers, veterinarians, and frontline health workers will be able to perform rapid and reliable anemia confirmatory screenings in field settings without the need for extensive laboratory infrastructure. This approach significantly streamlines the diagnostic process, reducing the time and expertise required to assess anemia in small ruminants, thereby potentially improving animal health management and treatment outcomes in agricultural settings.

One of the many ethical restrictions this study had to deal with was keeping the animals from becoming fatally ill, which restricted the range of PCV levels that could be recorded. Furthermore, the results may not be as generalizable as they may be due to the study’s reliance on a small sample size of 75 goats and lack of external validation, even if the study’s longitudinal approach produced an adequate dataset. To increase the robustness and usefulness of the generated models, future research should overcome these constraints by including external validation and larger sample sizes.

One limitation of this study is that image acquisition was conducted using only a single smartphone model, the Samsung A54. While the platform and application developed are designed to function on multiple devices, including both Android and iOS systems, the analytical variability associated with different smartphone models is currently unknown. Different devices may exhibit variations in image quality, such as resolution and lighting sensitivity, which could impact the consistency of results across platforms. Future studies should evaluate the performance of the application on a wider range of smartphone models to assess the generalizability and accuracy of the biosensor across different devices.

However, to mitigate potential discrepancies in image quality from various devices, the study employed several preprocessing steps, such as grayscale conversion and Otsu thresholding. These techniques focus on structural features of the images, reducing reliance on device-specific color or brightness variations. While these methods help standardize the images, further validation is needed to fully understand the extent of device variability and its potential impact on anemia detection accuracy.

The models evaluated in this study, including SVM, KNN, BPNN, and Keras Neural Network, demonstrated distinct limitations on the validation dataset that must be addressed for future improvements. The SVM model, while generally effective, showed reduced sensitivity for anemic animals and struggled with computational scalability, particularly for large datasets. Future improvements could include experimenting with more advanced kernel functions, addressing class imbalance using techniques like class weighting or SMOTE, and applying dimensionality reduction methods, such as PCA, to improve both training efficiency and model performance. The KNN model performed well in terms of sensitivity and specificity but was vulnerable to noisy data and computational inefficiencies due to the need to compute distances between every sample. Enhancements like data cleaning to reduce noise, using weighted KNN to emphasize nearer neighbors, and dimensionality reduction would make the KNN model more robust. The BPNN model was the best overall performer, achieving high scores across most metrics, but its deep architecture makes it computationally expensive and prone to overfitting without regularization. Regularization techniques, such as dropout and L2 regularization, combined with data augmentation and hyperparameter tuning, would help refine BPNN’s performance and prevent overfitting, especially with smaller datasets. Despite its promise, the Keras Neural Network underperformed for the dataset with a limited number of images, particularly for severely anemic animals, indicating issues with class imbalance, underfitting, and decision boundary calibration. To improve this model, future research should focus on refining the architecture, using longer training times with learning rate scheduling, incorporating class weighting or focal loss to handle imbalanced classes, and optimizing hyperparameters through grid search. Additionally, ensemble methods could be explored to combine the strengths of different models, while early stopping would help prevent overfitting in neural networks.

## 4 Conclusion and future research

This work introduces a new method for diagnosing anemia in goats by analyzing the fractal dimension of blood patterns as supported by a thorough assessment of different machine learning models. The fractal dimension measurements indicated that even in healthy goats, the blood patterns were intricate, with an average dimension of 0.87. This suggests that blood possesses fundamental textural characteristics that are connected to its natural viscosity and flow dynamics. As anemia worsens from mild to severe, the fractal dimension rose from 0.87 to 0.93, suggesting more noticeable modifications in texture caused by changes in blood characteristics. This technique holds potential for objectively evaluating the degree of anemia in animals by analyzing tiny alterations in the distribution of blood on filter paper saturated in glycerol.

Simultaneously, the effectiveness of four machine learning models, SVM, KNN, BPNN, and a Keras-based deep learning model, was evaluated at various stages of anemia in goats. The SVM model performed well, especially in accurately recognizing severe anemia with exact precision, recall, and F1 scores, suggesting its usefulness for clinical applications. The KNN model had difficulties when dealing with extremely anemic samples, most likely because of its susceptibility to imbalanced datasets. The BPNN and Keras models demonstrated strong performance, with the latter outperforming due to its deep learning capabilities that efficiently handled intricate patterns.

We have improved and evaluated a rapid blood biosensor for detecting anemia in small ruminants, as proposed by Laha et al. (30) for screening anemic human patients. It is more rapid than the traditional PCV measurement techniques used in veterinary health. Conventional PCV analysis requires laborious laboratory procedures and trained staff. On the other hand, our biosensor can deliver results within 5 min, providing a rapid and effective alternative. The capacity to conduct rapid tests not only speeds up the process of diagnosing diseases but also enables prompt decision-making, which is essential for efficiently managing the health of livestock.

In the future, this research has the potential to greatly influence both commercial and communal (Resource-Poor) farmers, not only in the USA, but also globally, by providing a readily available and cost-efficient method for early identification of anemia. By incorporating diagnostic tools into mobile platforms, farmers can complete routine health assessments on their animals without requiring specialized laboratory equipment or additional training, such as required for proper FAMACHA application. This has potential to significantly improve management and wellbeing of livestock, especially in distant or underserved regions with limited access to veterinary care. Moreover, continued progress in the model development could result not only in the utilization of this technology for small ruminants, but also for large ruminants and other domesticated animals, broadening its usefulness as an essential instrument for animal health assessment.

Future research could not only improve these methods by incorporating supplementary biomarkers, but also broaden the range of illnesses considered, specifically including the range of ubiquitous anemias, thus enhancing the diagnostic resources accessible to veterinarians. Therefore, this research not only enhances the comprehension of diagnostic procedures, but also creates new opportunities for rapid and dependable medical diagnostics in veterinary medicine, potentially improving the economic resilience and sustainability of farming operations globally.

## Data Availability

The raw data supporting the conclusions of this article will be made available by the authors, without undue reservation.

## Footnotes

^1^https://www.ibm.com/topics/knn
